# Dietary supplementation with *Bacillus*-based probiotic improves gut health in the weaned piglets challenged by rotavirus

**DOI:** 10.1186/s40104-025-01286-7

**Published:** 2025-11-29

**Authors:** Fengyu Xiang, Heng Yang, Xiangqi Fan, Dayan Tan, Bing Huang, Bing Yu, Jun He, Yuheng Luo, Junqiu Luo, Hui Yan, Junning Pu, Jianping Wang, Quyuan Wang, Huifen Wang, John Kyaw Htoo, Santa Maria Mendoza, Guiling Yan, Xiangbing Mao

**Affiliations:** 1 Animal Nutrition Institute, Sichuan Agricultural University, Key Laboratory for Animal Disease-Resistance Nutrition of China Ministry of Education, Key Laboratory of Animal Disease-Resistant Nutrition and Feed of China Ministry of Agriculture and Rural Affairs, Key Laboratory of Animal Disease-Resistant Nutrition of Sichuan Province, Chengdu, 611130 P.R. China; 2 Evonik Operations GmbH, Hanau-Wolfgang, 63457 Germany; 3 Evonik Corporation, Kennesaw, GA 30144 USA; 4 Evonik (China) Co., Ltd., Beijing, 100600 P.R. China

**Keywords:** Apoptosis, Antioxidant capacity, *Bacillus strains*, Intestinal health, Microbiota, Rotavirus, Weaned piglets

## Abstract

**Background:**

As probiotics, *Bacillus* strains may regulate some physiological functions in animals. This study aimed to evaluate whether dietary supplementation with a *Bacillus*-based probiotic could alleviate gut damage induced by rotavirus (RV) infection in piglets. Twenty-four piglets were randomly assigned into 2 groups fed with the basal diet (*n* = 16) and the diet containing 10^9^ colony-forming unit *Bacillus* spores/kg (*n* = 8). On d 8, 8 piglets fed with the diet supplemented with *Bacillus*-based probiotic and 8 piglets fed with basal diet were orally infused with RV, while the residue piglets had oral gavage of sterile essential medium. The trial duration was 12 d.

**Results:**

RV challenge induced diarrhea, significantly destroyed the morphology of jejunal mucosa (*P* < 0.05), significantly increased RV-antibody and RV non-structural protein 4 of jejunal mucosa (*P* < 0.05), significantly impaired antioxidant capacity (including malondialdehyde level, total antioxidant capacity and catalase activity), immunity (such as interleukin 2, interleukin 4 and secreted immunoglobulin A levels), mucins and the mRNA expression of tight-junction-related (such as Zonula occludens 1, occludin) and apoptotic-related (including B-cell lymphoma/leukaemia-2-associated X protein, B cell lymphoma/leukaemia-2, cysteinyl aspartate specific proteinases) genes of jejunal mucosa (*P* < 0.05), and, to some extents, affected the bacteria community structure and abundance of ileal digesta in piglets. However, *Bacillus*-based probiotic administration could significantly attenuate the negative effects of RV infection on gut health of piglets (*P* < 0.05).

**Conclusions:**

These findings suggested that supplementing *Bacillus*-based probiotic in the diet could decrease diarrhea rate, and improve gut health in weaned piglets, which was associated with regulating intestinal antioxidant capacity, apoptosis, and microbiota.

**Supplementary Information:**

The online version contains supplementary material available at 10.1186/s40104-025-01286-7.

## Background

Rotavirus (RV), known as a double-stranded virus, can easily infected young animals (including suckling and weaned pigs). It mainly replicates inside enterocytes, destroys gut integrity, and induces severe diarrhea, which will impair animal growth [1]. Although there are some vaccines for protecting piglets, the incidence rate of rotavirus-induced diarrhea is still high [2]. There is a need to develop nutritional strategy for prevention and treating of RV infections in pigs. Recent studies showed that some probiotics could bind surface proteins on RV, reduce its virulence, and relieve diarrhea in pigs [3, 4], which can efficiently prevent and/or treat RV infection and RV-induced damage in piglets [5–11].

*Bacillus* strains, known as probiotics, may regulate some physiological function of animals, including digestion and absorption, immune function, antioxidant capacity, and intestinal barriers [12–16]. And some in vitro studies also showed that *Bacillus subtilis* inhibited the infection of some viruses (such as viral hemorrhagic septicemia virus, influenza virus, and transmissible gastroenteritis virus) on cells [17–19]. However, it is unknown about the effect of *Bacillus* strain administration on RV infection.

In this study, we hypothesized that dietary supplementation with *Bacillus*-based probiotic could alleviate the negative effect of rotavirus challenge on growth and gut health of weaned piglets. Based on the mechanisms of RV inducing diarrhea, some possible ways of *Bacillus*-based probiotic were also preliminarily determined.

## Materials and methods

### Animals and diets

A total of 24 DLY (Duroc × Landrace × Yorkshire) weaned barrows (6.41 ± 0.14 kg) were individually housed in the metabolic cages (1.5 m × 0.7 m × 1.0 m), which were located in the temperature-controlled room (26 ± 2 °C). These piglets were fed with the experimental diets 4 times daily at 08:00, 12:00, 16:00 and 20:00, and provided with drinking water ad libitum. In the process of feeding each time, all piglets would sufficiently ingest their diets till they ceased intake.

The basal diet (i.e., CON diet) was formulated to satisfy the recommended nutrient requirements of pigs (7–11 kg) in National Research Council (NRC, 2012) [20] (Table 1). The test probiotic contains two *Bacillus* strains (DM 2763 and DM 3021) with the spore count of ≥ 2 × 10^9^ colony-forming unit (CFU)/g (GutPlus^®^ Virsorb supplied by Evonik Operations GmbH, Germany). The PRO diet was the basal diet supplemented with 10^9^ CFU *Bacillus* spores/kg.

**Table 1:** Composition of the basal diet (as-fed basis, %)

Ingredients	Contents
Corn	32.16
Soybean meal	10.47
Extruded corn	20.00
Extruded soybean	7.50
Wheat	11.00
High protein whey powder	7.50
Soy protein concentrate	5.00
Fish meal	2.00
L-Lysine·HCl	0.73
DL-Methionine	0.36
L-Threonine	0.33
L-Valine	0.19
L-Tryptophan	0.11
L-Isoleucine	0.08
Choline chloride (50%)	0.10
NaCl	0.30
Limestone	0.36
CaHPO_4_	1.45
Vitamin premix^1^	0.03
Phytase	0.01
Antioxidant	0.02
Mineral premix^2^	0.10
Zinc oxide	0.20
Total	100.00
Nutrient levels^3^	
ME, Mcal/kg	3.44
NE, Mcal/kg	2.58
Crude protein	19.54
SID^4^ lysine	1.35
SID methionine + cysteine	0.81
SID threonine	0.85
SID tryptophan	0.29
SID valine	0.92
SID isoleucine	0.75

^1^Vitamin premix provided the per kg of diet: 0.1 mg biotin, 15 mg pantothenic acid, 0.75 mg folic acid, 30 mg niacin, 9,000 IU VA, 3,000 IU VD_3_, 20 IU VE, 3 mg VK_3_, 1.5 mg VB_1_, 4 mg VB_2_, 3 mg VB_6_, 0.2 mg VB_12_

^2^Mineral premix provided the per kg of diet: 100 mg Fe (FeSO_4_·H_2_O), 100 mg Zn (ZnSO_4_·H_2_O), 20 mg Cu (CuSO_4_·5H_2_ O), 15 mg Mn (MnSO_4_·H_2_O), 0.3 mg I (KI), 0.3 mg Se (Na_2_SeO_3_)

^3^Nutrient levels were calculated values

^4^Standardized ileal digestible

### Experimental design and sample collection

Following 3 d of adaption period, all piglets were randomly allotted into two groups according to initial body weight, and were fed with the CON (*n* = 16) and PRO (*n* = 8) diets for 12 d, respectively. On d 8, all piglets were orally administrated 5 mL of the sterile 100 mmol/L sodium bicarbonate solution. Then, the piglets fed with PRO diet (PRO group, *n* = 8) and half of the piglets fed with CON diet (RV group, *n* = 8) were orally infused with 25 mL [1.33 × 10^–6^ tissue culture infective dose 50 (TCID_50_)/mL] of RV dissolved in the essential medium. The residue piglets (CON group, *n* = 8) had oral gavage of the same amount of sterile essential medium. After RV challenge, the diarrhea of all piglets was recorded daily. Fecal consistency was scored: 0, hard bar/hard granulous; 1, soft/forming; 2, dense/nor forming; 3, fluid/nor forming.

RV preparation and virus titre (TCID_50_) determination were carried out as described previously [21]. Briefly, RV (ATCC VR893) activated by 5 μg/mL trypsin (type IX, Sigma) for 30 min at 37 °C was inoculated with MA104 cells. Following 2 h of incubation at 37 °C, MA104 cells were washed three times with sterile PBS, and then incubated at 37 °C in Eagle minimal essential medium (MEM). When the extensive cytopathic effect was observed with microscope, the culture was frozen and thawed three times, and centrifuged at 3,000 × *g* for 10 min. The supernatant containing RV was stored at −80 °C. Then, MA104 cell was grown to 80%–90% confluence in 96-well plates, and infected with 50 μL aliquots of 1:10 serial dilutions (in MEM medium) of RV samples (8 wells/dilution). After the incubation for 4 d at 37 °C in 5% CO_2_, the cytopathic effect was visualized through staining the remaining viable cells with crystal violet. The virus titre (TCID_50_) was calculated.

On d 13, after fasting for 12 h, all piglets were weighed. The index of growth performance was calculated. Then, six piglets were randomly chosen. Blood samples were collected from jugular vein, centrifuged at 1,031 × *g* for 10 min, and serum was collected. Following intracardiac injection with sodium pentobarbital (50 mg/kg body weight) and jugular exsanguinations, the small intestine was removed. The jejunum and ileum were immediately separated. The segment (about 2 cm) of jejunum was fixed in 4% paraformaldehyde for the analysis of mucosal morphology. The residue of jejunum was used to gather mucosa via scraping gut wall with glass microscope slides. The digesta of ileum was also collected. The samples of jejunal mucosa and ileal digesta were quickly frozen in liquid nitrogen, and stored at −80 °C.

### Growth performance, serum urea nitrogen, and diarrhea

After weighing all piglets on d 1, 8 and 13, their body weight was recorded. And feed intake of all piglets was daily recorded. These were used to calculated body weight gain, averaged daily gain (ADG), average daily feed intake (ADFI), and/or feed conversion ratio (FCR). The level of serum urea nitrogen (SUN, Catalog No. C013-2-1) was measured by using the kits from Nanjing Jiancheng Bioengineering institute (Nanjing, China) and a BioTek Synergy HT microplate reader (BioTek Instruments, Winooski, VT, USA). Based on the diarrhea status after RV challenge, the diarrhea index was calculated as [(Σ fecal scores for duration of RV infusion)/n].

### Indices of RV infection in jejunal mucosa

About 100 mg of jejunal mucosa was added into 900 µL ice-cold saline solution, shattered at 4 °C, and then centrifuged at 1,031 × *g* for 15 min at 4 °C. The supernatants were used to the measurement of indices. The levels of RV antibody (RV-Ab, Catalog No. YX-182212P) and RV non-structural protein 4 (NSP4, Catalog No. YX-182214) in jejunal mucosa were detected with ELISA kits from Shanghai Nuoyuan Biotechnology Co., Ltd. (Shanghai, China) and a BioTek Synergy HT microplate reader (BioTek Instruments,Winooski, VT, USA).

### The permeability-related indices in serum

The activity of diamine oxidase (DAO, Catalog No. A088-2-1) in serum was measured through the kits from Nanjing Jiancheng Bioengineering Institute (Nanjing, China). Serum D-lactic acid level (Catalog No. MM-33732O1) was determined by using ELISA kits from Jiangsu Meimian Industrial Co., Ltd. (Nanjing, China). During measurement, a BioTek Synergy HT microplate reader (BioTek Instruments,Winooski, VT, USA) was used.

### Jejunal morphology

The jejunal morphology was determined as described previously [22]. Briefly, after fixing, the jejunal segment was embedded in paraffin. The consecutive section (5 μm) was stained with hematoxylin–eosin. In each jejunal sample, villus height and crypt depth from intact villi (at least 10) were determined at 40× magnification with an Olympus CK 40 microscope (Olympus Optical Company).

### Immune-related indices and mucins in jejunal mucosa

The samples of jejunal mucosa were prepared as above. The levels of mucin 1 (Catalog No. MM-77941O1), mucin 2 (Catalog No. 77506O1), interleukin 2 (IL-2, Catalog No. MM-0421O1), IL-4 (Catalog No. MM-0419O1), and secreted immunoglobulin A (sIgA, Catalog No. MM-36234O1) in jejunal mucosa were performed by ELISA kits from Jiangsu Meimian Industrial Co., Ltd. (Nanjing, China) and a BioTek Synergy HT microplate reader (BioTek Instruments,Winooski, VT, USA).

### mRNA expression of barrier-related and apoptotic-related genes in jejunal mucosa

Total RNA isolation, cDNA synthesis and real-time quantitative PCR were executed as described previously [23]. TRIzol reagent, RT reagents, SYBR Premix Ex Taq reagents, and genes’ primers listed in Table 2 were obtained from TaKaRa Biotechnology (Dalian) Co., Ltd. (Dalian, China). The mRNA expression of Zonula occludens 1 (*ZO-1*), Occludin, B cell lymphoma/leukaemia-2 (*Bcl-2*), B-cell lymphoma/leukaemia-2-associated X protein (*Bax*), cysteinyl aspartate specific proteinase 3 (*Caspase3*), *Caspase8*, *Caspase9*, and β-actin in jejunal mucosa were measured by QuantStudio 5 Real-Time PCR Detection System (ThermoFisher, Massachusetts, USA).

**Table 2:** Primer sequences used for real-time PCR

Genes	Nucleotide sequences of primers (5ˊ→3ˊ)
Occludin	F: CTACTCGTCCAACGGGAAAG
	R: ACGCCTCCAAGTTACCACTG
*ZO-1*	F: AAGCCCTAAGTTCAATCACAATCT
	R: ATCAAACTCAGGAGGCGGC
*Bax*	F: AAGCGCATTGGAGATGAACT
	R: TGCCGTCAGCAAACATTTC
*Bcl-2*	F: TGCCTTTGTGGAGCTGTATG
	R: GCCCGTGGACTTCACTTATG
*Caspase3*	F: GGGATTGAGACGGACAGTGG
	R: TGAACCAGGATCCGTCCTTTG
*Caspase8*	F: TCTGCGGACTGGATGTGATT
	R: TCTGAGGTTGCTGGTCACAC
*Caspase9*	F: AATGCCGATTTGGCTTACGT
	R: CATTTGCTTGGCAGTCAGGTT
β-Actin	F: TGGAACGGTGAAGGTGACAGC
	R: GCTTTTGGGAAGGCAGGGACT

### Antioxidant capacity in serum and jejunal mucosa

The samples of jejunal mucosa were prepared as above. The level of malondialdehyde (MDA, Catalog No. A003-1-2), the activities of catalase (CAT, Catalog No. A007-1-1) and superoxide dismutase (SOD, Catalog No. A001-3-2), and total antioxidant capacity (T-AOC, Catalog No. A015-1-2) in serum and jejunal mucosa were measured through the kits from Nanjing Jiancheng Bioengineering Institute (Nanjing, China) and a BioTek Synergy HT microplate reader (BioTek Instruments,Winooski, VT, USA).

### Ultra-high-throughput analysis of bacterial community and data analysis

Microbial DNA was isolated from ileal digesta samples using the E.Z.N.A.^®^ Stool DNA Kit (Omega Bio-tek, GA, USA) according to the manufacturer’s protocols. The sequencing was executed by Shanghai Biozeron Biotechnology Co., Ltd. (Shanghai, China). The V3–V4 region of the bacteria 16S ribosomal RNA gene were amplified by PCR (95 °C for 2 min, followed by 27 cycles at 95 °C for 30 s, 55 °C for 30 s, and 72 °C for 60 s and a final extension at 72 °C for 5 min) using primers (341F: 5′-CCTAYGGGRBGCASCAG-3′, and 806R: 5′-GGACTACNNGGGTATCTAAT-3′). PCR reactions were performed in triplicate 20 µL mixture containing 4 µL of 5 × FastPfu Buffer, 2 µL of 2.5 mmol/L dNTPs, 0.8 µL of each primer (5 µmol/L), 0.4 µL of FastPfu Polymerae, and 10 ng of template DNA. Amplicons were extracted from 2% agarose gels and purified using the AxyPrep DNA Gel Extraction Kit (Axygen Biosciences, CA, USA) according to the manufacturer’s instructions. Operational taxonomical units (OTUs) were clustered with 97% similarity cutoff using UPARSE (version 7.1, http://drive5.com/uparse/), and chimeric sequences were identified and removed with UCHIME. The phylogenetic affiliation of each 16S rRNA gene sequence was analyzed by RDP Classifier (http://rdp.cme.msu.edu/) against the SILVA (SSU 132) 16S rRNA database using the confidence threshold of 70% [24]. The sequencing data were processed using Mothur v1.21.1 software (MI, USA) to obtain after quality control. The rarefaction analysis was conducted to reveal the diversity indices, such as Chao 1, ACE, and Simpson diversity indices. The beta diversity analysis was performed using Weighted UniFrac for principal coordinate analysis (PCoA) through QIIME (version 1.9.1) in vegan R package (version 4.1.2). The relative abundance of different bacterial taxa was expressed as percentages, which was used to analyze bacterial composition at the phylum and genus levels.

### Statistical analysis

All experimental data were analyzed by using SPSS 22.0 (IBM Corp., Armonk, NY, USA), and results were represented as means ± SE (standard error). (i) The data before RV challenge was analyzed by unpaired *t*-test (SPSS 22.0) if the data were in Gaussian distribution and had equal variance. (ii) Following RV challenge, the data between CON group and RV group, as well the data between RV group and PRO group, were analyzed with the unpaired *t*-test (SPSS 22.0) if the data were in Gaussian distribution and had equal variance. The difference (*P* < 0.05) was considered statistical significance.

## Results

### Growth performance, serum urea nitrogen level, and diarrhea of piglets

**Fig. 1:**
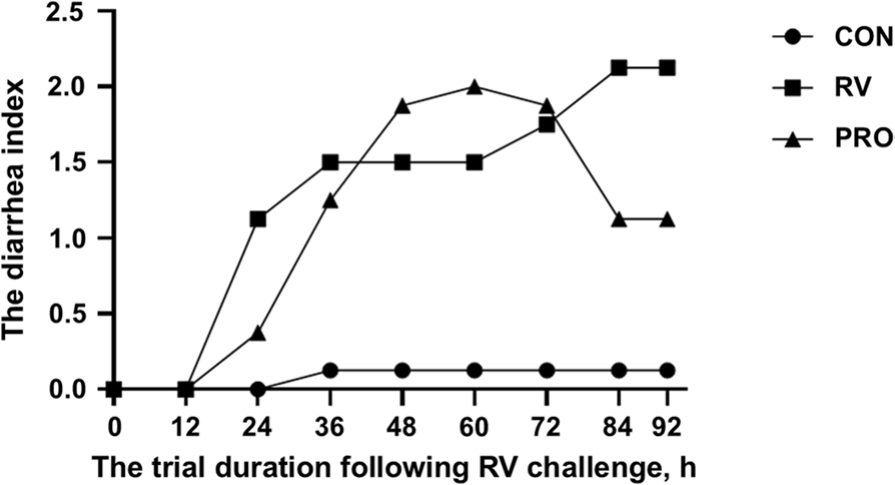
The diarrhea index of piglets after RV challenge. CON, basal diet; RV, basal diet + RV challenge; PRO, the diet supplemented *Bacillus*-based probiotic + RV challenge

As shown in Table 3, before oral gavage with RV (challenge), dietary probiotic supplementation did not significantly affect growth performance of piglets (*P* > 0.05). During post-challenge (d 8–12), RV challenge decreased body weight gain of piglets, which could be alleviated by probiotic administration (25% higher than RV), but these effects had not significant differences (*P* > 0.05, Table 3). And *Bacillus*-based probiotic administration significantly decreased SUN level in the RV-infected piglets (*P* < 0.05, Table 3). In addition, inoculation with RV induced diarrhea of piglets, and supplementing *Bacillus*-based probiotic in the diet could reduce the severity of diarrhea at 84 h following RV challenge (Fig. 1).

**Table 3:** Effects of dietary *Bacillus*-based probiotic supplementation on growth performance and serum urea nitrogen level of weaned piglets with or without rotavirus challenge

Item	CON	RV	PRO
1–7 d (Pre-challenge)			
ADG, g/d	154.1 ± 22.3		138.4 ± 39.7
ADFI, g/d	207.5 ± 24.8		212.6 ± 56.8
FCR, g/g	1.43 ± 0.08		1.61 ± 0.14
8–12 d (Post-challenge)			
Body weight gain, g	873.8 ± 256.8	431.3 ± 297.9	538.8 ± 371.9
ADFI, g/d	314.3 ± 76.3	359.0 ± 89.8	313.6 ± 41.7
SUN, mmol/L	6.78 ± 1.21	9.62 ± 1.44	6.02 ± 0.82^*^

1–7 d: CON, basal diet (*n* = 16); PRO, the diet supplemented *Bacillus*-based probiotic (*n* = 8). 8–12 d: CON, basal diet (*n* = 8); RV, basal diet + RV challenge (*n* = 8); PRO, the diet supplemented *Bacillus*-based probiotic + RV challenge (*n* = 8)

*ADG* Average daily gain, *ADFI* Average daily feed intake, *FCR* Feed conversion ratio, *SUN* Serum urea nitrogen

^*^Compared with RV group, the value had significant difference (*P* < 0.05, *n* = 6)

### The indices of RV infection

As shown in Table 4, RV challenge significantly enhanced the concentrations of RV-Ab and NSP4 in the jejunum of weaned piglets (*P* < 0.05). Compared with the piglets of RV group, those of PRO group had lower RV-Ab and NSP4 levels in the jejunum, but this effect was not significant (*P* > 0.05, Table 4).

**Table 4:** Effects of dietary *Bacillus-*based probiotic supplementation on rotavirus infection in the jejunum of weaned piglets with or without rotavirus challenge at d 4 post-challenge

Item	CON	RV	PRO
RV-Ab, μg/mg protein	2.19 ± 0.18	3.16 ± 0.14^#^	2.68 ± 0.29
NSP4, ng/mg protein	4.74 ± 0.65	9.00 ± 0.49^#^	7.78 ± 0.98

CON, basal diet; RV, basal diet + RV challenge; PRO, the diet supplemented *Bacillus*-based probiotic + RV challenge

*RV-Ab* Rotavirus antibody, *NSP4* Non-structural protein 4

^#^Compared with CON group, the value had significant difference (*P* < 0.05, *n* = 6)

### Gut-barrier-related indices

Compared with the piglets of CON group, those of RV group had higher D-lactic acid level, and DAO activity in serum (*P* < 0.05, Table 5). However, serum D-lactic acid level and DAO activity in the piglets of PRO group were significantly lower than those in the piglets of RV group (*P* < 0.05, Table 5).

**Table 5:** Effects of dietary *Bacillus-*based probiotic supplementation on gut-barrier-related indices in the serum and jejunum of weaned piglets with or without rotavirus challenge at d 4 post-challenge

Item	CON	RV	PRO
The permeability-related indices in serum			
D-Lactic acid, mmol/mL	139.20 ± 7.68	224.60 ± 9.35^#^	133.60 ± 8.67^*^
DAO, U/L	8.52 ± 0.45	13.62 ± 0.77^#^	5.06 ± 0.82^*^
Jejunal morphology			
Villus height, μm	396.90 ± 35.43	263.20 ± 11.63^#^	350.60 ± 44.00
Crypt depth, μm	235.80 ± 15.90	354.70 ± 17.52^#^	262.90 ± 25.81^*^
Villus height/crypt depth	1.70 ± 0.08	0.76 ± 0.04^#^	1.38 ± 0.09^*^
Mucins in jejunum			
Mucin 1, μg/mg protein	0.99 ± 0.05	0.59 ± 0.03^#^	0.90 ± 0.06^*^
Mucin 2, μg/mg protein	0.70 ± 0.04	0.40 ± 0.04^#^	0.63 ± 0.03^*^
The expression of barrier-related genes in jejunum			
*ZO-1*	1.00 ± 0.18	0.29 ± 0.05^#^	1.31 ± 0.25^*^
Occludin	1.00 ± 0.24	0.29 ± 0.06^#^	1.15 ± 0.22^*^
The immune-related indices in jejunum			
IL-2, ng/mg protein	0.43 ± 0.04	0.59 ± 0.03^#^	0.42 ± 0.05^*^
IL-4, μg/mg protein	0.93 ± 0.06	0.54 ± 0.03^#^	0.79 ± 0.07^*^
sIgA, μg/mg protein	0.35 ± 0.02	0.25 ± 0.03^#^	0.34 ± 0.03^*^

CON, basal diet; RV, basal diet + RV challenge; PRO, the diet supplemented *Bacillus*-based probiotic + RV challenge

*DAO* Diamine oxidase, *ZO-1* Zonula occludens 1, *IL-2* Interleukin 2, *IL-4* Interleukin 4, *sIgA* Secreted immunoglobulin A

^#^Compared with CON group, the value had significant difference (*P* < 0.05, *n* = 6)

^*^Compared with RV group, the value had significant difference (*P* < 0.05, *n* = 6)

**Fig. 2:**
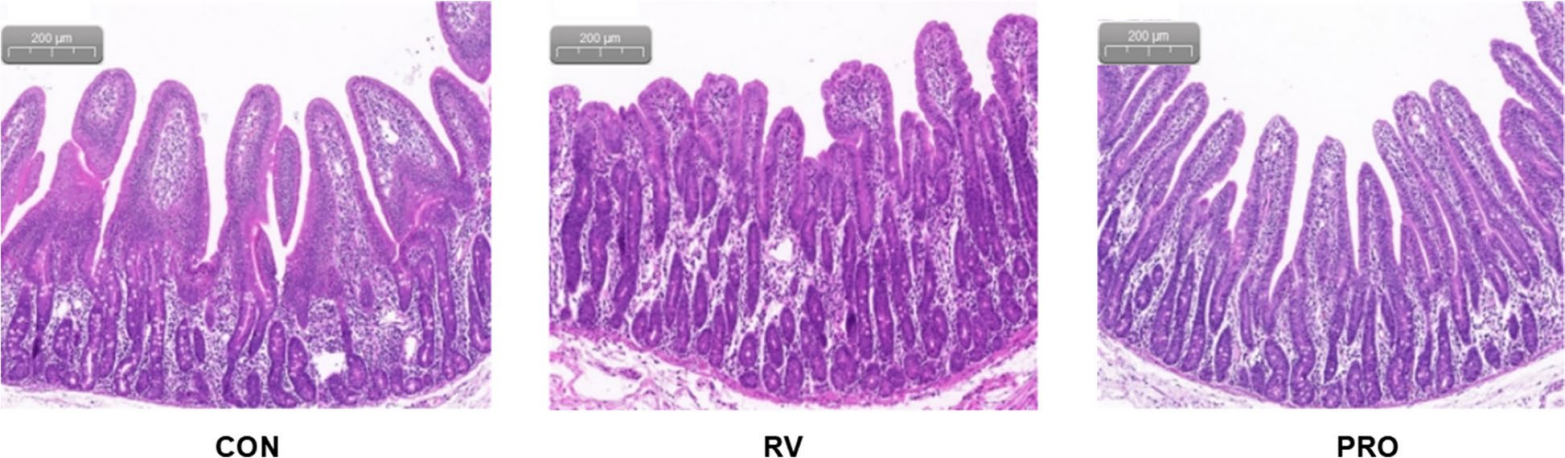
The histology of jejunal mucosa in weaned piglets. CON, basal diet; RV, basal diet + RV challenge; PRO, the diet supplemented *Bacillus*-based probiotic + RV challenge

Oral inoculation with RV destroyed the villus morphology (Fig. 2), significantly reduced villus height and villus height/crypt depth (*P* < 0.05, Table 5), and significantly increased crypt depth (*P* < 0.05, Table 5) in the jejunal mucosa of weaned piglets. When the piglets of PRO group were compared with those of RV group, *Bacillus*-based probiotic administration effectively attenuated the RV-induced damage of jejunal villi (Fig. 2), significantly decreased crypt depth (*P* < 0.05, Table 5), and significantly enhanced villus height/crypt depth (*P* < 0.05, Table 5) in the jejunum.

As shown in Table 5, RV infection significantly enhanced IL-2 concentration, and significantly reduced the contents of IL-4, sIgA, mucin 1 and mucin 2, and significantly downregulated the mRNA expression of *ZO-1* and Occludin in the jejunum of weaned piglets (*P* < 0.05). Compared with the piglets of RV group, those of PRO group had higher IL-4, sIgA, mucin 1 and mucin 2 levels, and the mRNA expression of *ZO-1* and Occludin, as well had lower IL-2 content in the jejunum of weaned piglets (*P* < 0.05, Table 5).

### The mRNA expression of apoptotic-related genes

Compared with the piglets of CON group, the piglets of RV group had the lower mRNA expression of *Bcl-2*, and the higher mRNA expression of *Bax*, *Caspase3*, *Caspase8* and *Caspase9* in the jejunal mucosa (*P* < 0.05, Table 6). When the piglets of PRO group were compared with those of RV group, *Bacillus*-based probiotic supplementation significantly upregulated the mRNA expression of *Bcl-2*, and downregulated the mRNA expression of *Bax*, *Caspase8* and *Caspase9* in the jejunal mucosa (*P* < 0.05, Table 6).

**Table 6:** Effects of dietary *Bacillus-*based probiotic supplementation on the expression of apoptotic-related genes in the jejunum of weaned piglets with or without rotavirus challenge at d 4 post-challenge

Item	CON	RV	PRO
*Bax*	1.00 ± 0.18	2.78 ± 0.38^#^	1.11 ± 0.28^*^
*Bcl-2*	1.00 ± 0.11	0.55 ± 0.10^#^	1.17 ± 0.13^*^
*Caspase3*	1.00 ± 0.13	1.67 ± 0.17^#^	1.20 ± 0.19
*Caspase8*	1.00 ± 0.14	2.56 ± 0.23^#^	1.55 ± 0.17^*^
*Caspase9*	1.00 ± 0.20	2.84 ± 0.24^#^	1.98 ± 0.20^*^

CON, basal diet; RV, basal diet + RV challenge; PRO, the diet supplemented *Bacillus*-based probiotic + RV challenge

*Bcl-2* B cell lymphoma/leukaemia-2, *Bax* B-cell lymphoma/leukaemia-2-associated X protein, *Caspase* Cysteinyl aspartate specific proteinase

^#^Compared with CON group, the value had significant difference (*P* < 0.05, *n* = 6)

^*^Compared with RV group, the value had significant difference (*P* < 0.05, *n* = 6)

### Antioxidant capacity of serum and jejunum

Effects of dietary *Bacillus-*based probiotic supplementation on antioxidant capacity in the serum and jejunum of weaned piglets with or without rotavirus challenge at d 4 post-challenge were shown in Table 7. In the weaned piglets, RV infection significantly enhanced the MDA concentration of serum and jejunum, as well significantly reduced CAT activity of jejunum, and T-AOC, SOD and CAT activities of serum (*P* < 0.05). Compared with the piglets of RV group, those of PRO group had higher T-AOC and CAT activity of jejunum and serum, as well had lower MDA content of jejunum and serum (*P* < 0.05).

**Table 7:** Effects of dietary *Bacillus-*based probiotic supplementation on antioxidant capacity in the serum and jejunum of weaned piglets with or without rotavirus challenge at d 4 post-challenge

Item	CON	RV	PRO
Serum			
MDA, mmol/mL	2.98 ± 0.73	8.28 ± 0.89^#^	4.38 ± 0.35^*^
T-AOC, U/mL	2.85 ± 0.57	1.35 ± 0.12^#^	2.33 ± 0.37^*^
SOD, U/mL	75.86 ± 2.97	67.38 ± 1.46^#^	70.26 ± 3.36
CAT, U/mL	9.13 ± 1.00	5.00 ± 0.68^#^	7.82 ± 0.58^*^
Jejunum			
MDA, mmol/mg protein	0.29 ± 0.03	0.82 ± 0.09^#^	0.33 ± 0.02^*^
T-AOC, U/mg protein	0.50 ± 0.06	0.38 ± 0.05	0.66 ± 0.09^*^
SOD, U/mg protein	10.37 ± 0.25	9.75 ± 0.21	9.66 ± 0.10
CAT, U/mg protein	9.71 ± 0.66	4.81 ± 0.59^#^	8.59 ± 1.10^*^

CON, basal diet; RV, basal diet + RV challenge; PRO, the diet supplemented *Bacillus*-based probiotic + RV challenge

*MDA* Malondialdehyde, *T-AOC* Total antioxidant capacity, *SOD* Superoxide dismutase, *CAT* Catalase

^#^Compared with CON group, the value had significant difference (*P* < 0.05, *n* = 6)

^*^Compared with RV group, the value had significant difference (*P* < 0.05, *n* = 6)

### Bacterial community structure in the ileal digesta

**Fig. 3:**
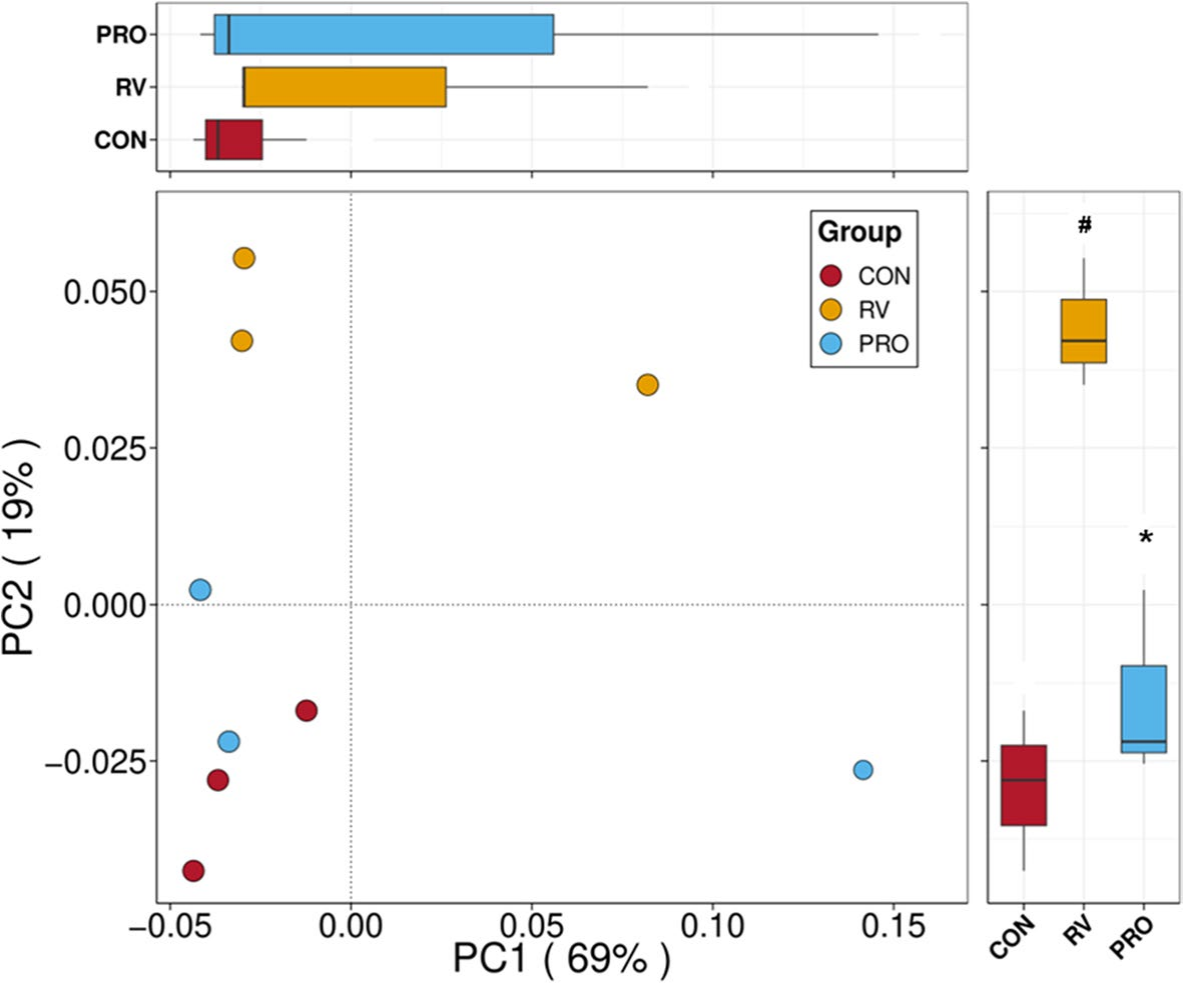
PCoA of bacterial community structures in the ileal digesta of weaned piglets. CON, basal diet; RV, basal diet + RV challenge; PRO, the diet supplemented *Bacillus*-based probiotic + RV challenge. ^#^Compared with CON group, the value had significant difference (*P* < 0.05, *n* = 3). ^*^Compared with RV group, the value had significant difference (*P* < 0.05, *n* = 3)

The alpha diversity indices (including Chao 1, ACE, and Simpson) in the ileal digesta of pigs were shown in Fig. S1. There were no significant differences in the alpha diversity index of ileal digesta between CON and RV groups, and between RV and PRO groups (*P* > 0.05). And the PCoA of weighted UniFrac distances showed that there was a significant difference between the communities of CON and RV groups (*P* < 0.05, Fig. 3), and this effect of RV infection was significantly alleviated by dietary probiotic supplementation (*P* < 0.05, Fig. 3).

**Fig. 4:**
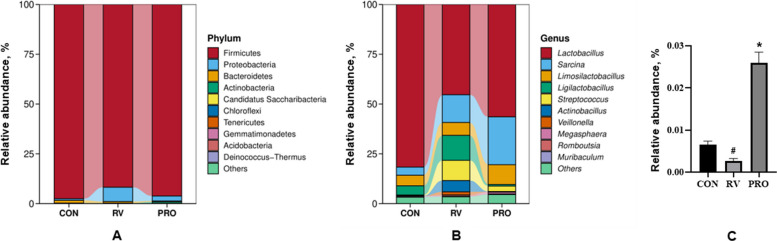
The relative abundance of bacterial composition. **A** The phylum level. **B** The genus level. **C** Relative abundance of *B. subtilis*. CON, basal diet; RV, basal diet + RV challenge; PRO, the diet supplemented *Bacillus*-based probiotic + RV challenge. ^#^Compared with CON group, the value had significant difference (*P* < 0.05, *n* = 3). ^*^Compared with RV group, the value had significant difference (*P* < 0.05, *n* = 3)

At the phylum level, the dominant phyla in the ileal digesta of pigs were Firmicutes and Proteobacteria, followed by Bacteroidetes, Actinobacteria and Candidatus Saccharibacteria (Fig. 4A). Of these, the abundance of Proteobacteria in RV group only tended to be higher than that in CON group (7.20% vs. 0.81%, *P* = 0.08), and the abundance of Proteobacteria in PRO group was lower than that in RV group (2.49% vs. 7.20%, *P* = 0.24) (Table S1).

At the genus level, the prominent differences of genera in the ileal digesta of pigs were shown in Fig. 4B and Table S2. The dominant genera in the ileal digesta of pigs were *Lactobacillus*, *Sarcina*, *Limosilactobacillus*, *Ligilactobacillus*, *Streptococcus*, and *Actinobacillus*. Of these, RV challenge significantly decreased the abundance of *Lactobacillus* (*P* < 0.05), significantly boosted the abundance of *Streptococcus* (*P* < 0.05), and tended to enhance the abundance of *Actinobacillus* in the ileal digesta of piglets (*P* = 0.10). When the piglets of PRO group were compared with those of RV group, dietary *Bacillus*-based probiotic supplementation could diminish the abundance of *Ligilactobacillus* (*P* < 0.05), *Streptococcus* (*P* < 0.05), and *Actinobacillus* (*P* = 0.10) in the ileal digesta.

In addition, the abundance of *Bacillus subtilis* (*B. subtilis*) in the ileal digesta of piglets was also detected. As shown in Fig. 4C, the abundance of *B. subtilis* in RV group was significantly lower than that in CON group (*P* < 0.05), but the abundance of *B. subtilis* in PRO group was significantly higher than that in RV group (*P* < 0.05).

## Discussion

Pathogenic diarrhea severely impairs swine breeding. It is well-known that RV is one of the main pathogens that induce diarrhea and gut damage in piglets [1]. Some studies (including previous ours) have shown that RV infection will lead to diarrhea and the decrease of growth performance, destroy the intestinal morphology, and have the negative effect on immunity, non-specific barrier functions, antioxidant capacity and gut microbiota in pigs [1, 25, 26]. The current study also got the similar results, and further found that RV-Ab and NSP4 levels were significantly increased in the jejunal mucosa of piglets. RV-Ab is the specific antibody against RV that is produced by animals and human infected by RV, and NSP4 is one of the nonstructural proteins of RV [1]. Therefore, the RV-infected model was successfully established in piglets.

This study showed that although the difference was nonsignificant, compared with RV group, body weight gain of pigs in PRO group was increased by closely 25%. In addition, dietary probiotic supplementation decreased SUN level of piglets. Urea in blood indirectly embodies in the utilization of nutrients (especially protein) in whole body, which is often considered as the index of growth performance [27]. Many previous studies have also shown that some probiotics (such as Lactobacilli, Bifidobacteria, *Escherichia coli* Nissle 1917) also exists the similar improvement to growth of piglets challenged by RV [5–11]. Thus, probiotic administration have the potential to improve the growth performance of RV-infected pigs.

Gut plays a critical role in growth and health of animals, which is also the main organ of RV invasion and infection [1, 28]. Non-specific barrier functions are very important to intestinal health, which are composed of mucosal integrity, mucus gel layer and tight junctions [29]. The morphology can reflect mucosal integrity, mucins are the primary content of mucus gel layer, and some proteins (such as ZO-1, Occludin) are the constituents of tight junctions [30–32]. Moreover, serum D-lactic acid level and DAO activity may be considered as the index of intestinal permeability [33, 34]. In our study, RV challenge impaired morphology, the levels of mucin 1 and 2, and the mRNA expression of *ZO-1* and Occludin in the jejunal mucosa, and serum D-lactic acid level and DAO activity of piglets, thereby the negative effects were alleviated by dietary probiotic supplementation. These demonstrated that the administration of *Bacillus*-based probiotic should be helpful for maintaining gut health in the RV-infected pigs.

NSP4, known as a kind of non-structural protein in RV, is the key factor of RV invasion, and may reflect the status of RV infection [2]. Our study showed that probiotic administration numerically (−14%) decreased NSP4 level in the jejunal mucosa of RV-infected pigs, but it was not significant. This indicated that although there were the increasing sIgA and mucin levels in jejunal mucosa, the effect of *Bacillus*-based probiotic on reducing gut damage may not be mainly due to RV prevention or clearance.

Gut health is closely related to the survival of epithelial cells [35]. RV infection can upregulate the apoptosis of epithelial cells [2]. In this study, the apoptotic-related genes (*Bcl-2*, *Bax*, *Caspase3*, *Caspase8*, and *Caspase9*) were measured, and supplementing* Bacillus*-based probiotic in the diet downregulated the mRNA expression of *Bax*, *Caspase8* and *Caspase9*, and upregulated the mRNA expression of *Bcl-2* in the jejunal mucosa of RV-challenged pigs.

Redox balance and inflammation are the important factors of influencing cell apoptosis [36, 37]. In the process of RV infection, redox balance of epithelial cells will be destroyed, and antioxidant capacity is inhibited [2]. At 3–4 d following RV challenge, excessive inflammation exists in intestines [2]. In this study, dietary *Bacillus*-based probiotic supplementation stimulated IL-4 level, T-AOC and CAT activity, and inhibited IL-2 and MDA levels in the jejunal mucosa of RV-infected pigs. Some in vivo and in vitro researches have also found that the probiotics (i.e. *Lactobacillus rhamnosus* GG, *Bacillus clausii*) might improve antioxidant capacity and/or inflammation, and then increase the survival of gut epithelial cells challenged by RV [3, 38–40]. Thus, it is possible that the administration of probiotics (including *Bacillus*-based probiotic) alleviating the cell apoptosis induced by RV invasion should be derived from the improvement of antioxidant capacity and/or inflammation.

Gut microbiota can affect animal health (especially gut health) [41]. The previous studies in human (e.g., infants) reported that RV infection can change gut microbiota formation [25, 42]. In this study, PCoA analysis of weighted UniFrac showed that there were differences of bacteria composition in the ileal digesta among 3 groups, and then RV infection obviously changed the bacteria composition, which was weakened by probiotic administration. These primarily illustrated that dietary *Bacillus*-based probiotic supplementation could improve bacteria community structure in the RV-infected piglets. However, there were some differences between this study and the previous ones, which could be mainly due to the diversity of host species.

In the previous studies, the further analysis found that RV infection can change the abundance of some bacteria (e.g., Proteobacteria) in the intestine of humans [25, 42], which is similar with our results. In Proteobacteria, *Streptococcus*, and *Actinobacillus*, many members can be the cause of intestinal inflammation and injury in humans [43–45]. And the current study showed that *Bacillus*-based probiotic administration, to some extents, diminished these bacteria in the ileal digesta of piglets, which would possibly be beneficial for gut health. Thereby, *Bacillus*-based probiotic had the potential to improve gut microbiota impaired by RV infection in pigs. In the ileal digesta of PRO-group pigs, the abundance of *B. subtilis* was significantly increased, which could verify the success of supplementing *Bacillus*-based probiotic in the diet.

## Conclusions

In summary, RV infection induced diarrhea and gut damage in weaned piglets, which might efficiently be attenuated by dietary supplementation with *Bacillus*-based probiotic (GutPlus^®^ Virsorb). Further analysis found that the effect of *Bacillus*-based probiotic should be associated with the downregulation of gut apoptosis via improving antioxidant capacity and inflammation, and the improvement of gut microbiota in pigs. These results will further expand the mechanism of *Bacillus*-based probiotic ameliorating gut health, and be beneficial for the application of *Bacillus*-based probiotic relieving diarrhea (especially RV-induced diarrhea) in swine production.

## Supplementary Information

Additional file 1: Fig. S1. The bacterial community diversity and richness.

Additional file 2: Table S1. Effects of dietary *Bacillus subtilis* supplementation on the relative abundance of dominant phylum in the ileal digesta of weaned piglets with or without rotavirus challenge at d 4 post-challenge. Table S2. Effects of dietary *Bacillus subtilis* supplementation on the relative abundance of dominant genus in the ileal digesta of weaned piglets with or without rotavirus challenge at d 4 post-challenge.

## Data Availability

The datasets used and/or analyzed during the current study are available from the corresponding author on reasonable request.

## Abbreviations

ADFI: Average daily feed intake
ADG: Average daily gain
Bax: B-cell lymphoma/leukaemia-2-associated X protein
Bcl-2: B cell lymphoma/leukaemia-2
*B. subtilis*: *Bacillus subtilis*
Caspase3: Cysteinyl aspartate specific proteinase 3
Caspase8: Cysteinyl aspartate specific proteinase 8
Caspase9: Cysteinyl aspartate specific proteinase 9
CAT: Catalase
CFU: Colony-forming unit
DAO: Diamine oxidase
FCR: Feed conversion ratio
IL-2: Interleukin 2
IL-4: Interleukin 4
MDA: Malondialdehyde
NSP4: Non-structural protein 4
PCoA: Principal coordinate analysis
RV: Rotavirus
RV-Ab: Rotavirus antibody
sIgA: Secreted immunoglobulin A
SOD: Superoxide dismutase
SUN: Serum urea nitrogen
TCID_50_: Tissue culture infective dose 50
T-AOC: Total antioxidant capacity
ZO-1: Zonula occludens 1
