# Supplementary figures and images for: Dietary supplementation with Bacillus-based probiotic improves gut health in the weaned piglets challenged by rotavirus
Source: J Anim Sci Biotechnol. 2025 Nov 29;16:161. doi: 10.1186/s40104-025-01286-7 (PMC12664204; doi:10.1186/s40104-025-01286-7)

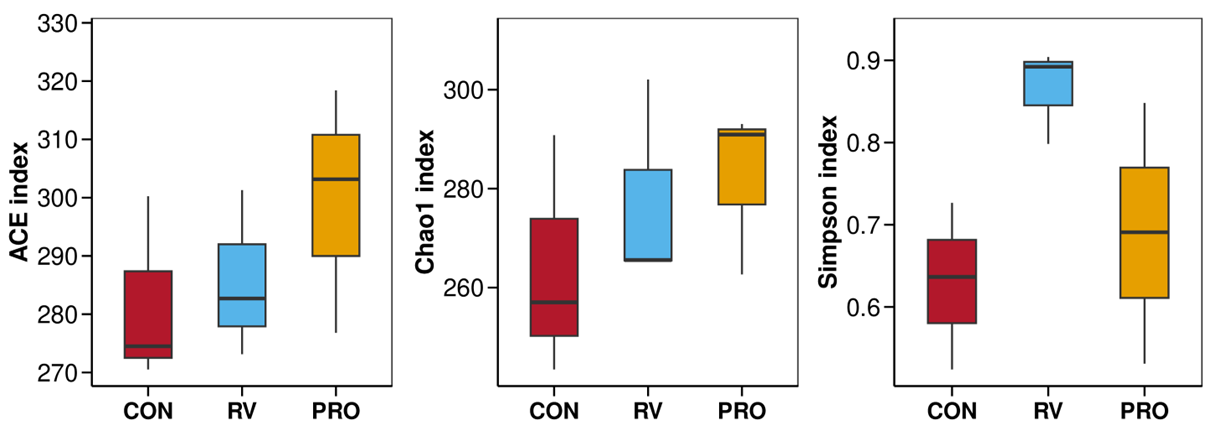

Supplement: Supplementary file 1 — Additional file 1: Fig. S1. The bacterial community diversity and richness. [file 40104_2025_1286_MOESM1_ESM.tif]
