# Supplementary material for: Dietary supplementation with Bacillus-based probiotic improves gut health in the weaned piglets challenged by rotavirus
Source: J Anim Sci Biotechnol. 2025 Nov 29;16:161. doi: 10.1186/s40104-025-01286-7 (PMC12664204; doi:10.1186/s40104-025-01286-7)
Supplement: Supplementary file 2 — Additional file 2: Table S1. Effects of dietary Bacillus subtilis supplementation on the relative abundance of dominant phylum in the ileal digesta of weaned piglets with or without rotavirus challenge at d 4 post-challenge. Table S2. Effects of dietary Bacillus subtilis supplementation on the relative abundance of dominant genus in the ileal digesta of weaned piglets with or without rotavirus challenge at d 4 post-challenge. [file 40104_2025_1286_MOESM2_ESM.docx]

**Table S1** Effects of dietary *Bacillus subtilis* supplementation on the relative abundance of dominant phylum in the ileal digesta of weaned piglets with or without rotavirus challenge at d 4 post-challenge

|  | **CON** | **RV** | **PRO** |
| --- | --- | --- | --- |
| Firmicutes | 97.55 ± 1.25 | 91.77 ± 2.81 | 96.17 ± 2.56 |
| Proteobacteria | 0.81 ± 0.21 | 7.20 ± 2.73 | 2.49 ± 2.02 |

CON, basal diet (*n* = 3); RV, basal diet + RV challenge (*n* = 3); PRO, the diet supplemented *Bacillus*-based probiotic + RV challenge (*n* = 3)

**Table S2** Effects of dietary *Bacillus subtilis* supplementation on the relative abundance of dominant genus in the ileal digesta of weaned piglets with or without rotavirus challenge at d 4 post-challenge

|  | **CON** | **RV** | **PRO** |
| --- | --- | --- | --- |
| *Lactobacillus* | 81.68 ± 7.41 | 45.34 ± 6.31^#^ | 56.46 ± 16.69 |
| *Sarcina* | 4.07 ± 3.58 | 13.95 ±12.51 | 24.03 ± 21.77 |
| *Limosilactobacillus* | 5.31 ± 2.18 | 6.47 ± 2.61 | 9.90 ± 6.91 |
| *Ligilactobacillus* | 4.64 ± 2.49 | 12.52 ± 3.09 | 0.82 ± 0.24^*^ |
| *Streptococcus* | 0.32 ± 0.15 | 10.15 ± 0.86^#^ | 2.71 ± 2.53^*^ |
| *Actinobacillus* | 0.10 ± 0.04 | 5.68 ± 2.62 | 0.15 ± 0.09 |

CON, basal diet; RV, basal diet + RV challenge; PRO, the diet supplemented *Bacillus*-based probiotic + RV challenge

^#^Compared with CON group, the value had significant difference (*P* < 0.05, *n* = 3)

^*^Compared with RV group, the value had significant difference (*P* < 0.05, *n* = 3)
